# Preoperative diagnosis of *BRCA1/2* mutation impacts decision-making for risk-reducing mastectomy in breast cancer patients

**DOI:** 10.1038/s41598-021-94195-4

**Published:** 2021-07-20

**Authors:** Jinsun Woo, Geumhee Gwak, Inseok Park, Byung Noe Bae, Se Kyung Lee, Byung Joo Chae, Jonghan Yu, Jeong Eon Lee, Seok Won Kim, Seok Jin Nam, Jai Min Ryu

**Affiliations:** 1 Division of Breast Surgery, Department of Surgery, Samsung Medical Center Cancer Hospital, Sungkyunkwan University School of Medicine, 5th Floor Breast-Endocrine, 81 Irwon-ro, Gangnam-gu, Seoul, 06351 South Korea; 2 Department of Surgery, Sanggye Paik Hospital, Inje University College of Medicine, Seoul, South Korea

**Keywords:** Cancer, Surgical oncology, Genetic testing

## Abstract

Decision to undergo risk-reducing mastectomy (RRM) needs to consider several factors, including patient’s preference, surgeon’s preference, family history, and genetic predisposition. The aim of this study was to examine whether preoperative diagnosis of *BRCA1/2* mutation status could influence surgical decision-making in newly diagnosed breast cancer patients. We retrospectively reviewed ipsilateral breast cancer patients with *BRCA1/2* mutation who underwent primary surgery between January 2008 and November 2019 at a single institution in Korea. Of 344 eligible patients, 140 (40.7%) patients were aware of their mutation status ‘prior to surgery’, while 204 (59.3%) did not. Contralateral RRM rate was significantly higher in the group with *BRCA1/2* mutation status identified ‘prior to surgery’ compared to the group with mutation status identified ‘after surgery’ [45.0% (63/140) vs. 2.0% (4/204)] (*p* < 0.001). Reduced turnaround time of *BRCA1/2* testing (*p* < 0.001) and the use of neoadjuvant chemotherapy (*p* < 0.001) were associated with *BRCA1/2* mutation status identified prior to surgery. Although not statistically significant, higher incidence of developing contralateral breast cancer for *BRCA1/2* mutation carriers who underwent ipsilateral surgery-only compared to those who underwent contralateral RRM was observed [12.1% (95% CI: 7.7–17.7%)] (*p* = 0.1618). Preoperative diagnosis of *BRCA1/2* mutation could impact surgical decision-making for breast cancer patients to undergo risk-reducing surgery at the time of initial surgery.

## Introduction

A celebrity’s announcement of having a *BRCA1* mutation and undergoing bilateral risk-reducing mastectomy (RRM) has influenced breast cancer patients and women with high-risk breast cancer. The so-called “Angelina’s effect” has impacted public’s health awareness of hereditary breast cancer and increased rates of genetic counseling and tests for *BRCA1/2* mutations^1–3^. This has also led to an increase in the rate of RRM among women with inherited *BRCA1/2* mutation.

*BRCA1/2* mutation carriers who have developed breast cancer have a significant higher risk for ipsilateral breast recurrence with a median follow-up ≥ 7 years compared to non-carriers^4^. These patients also have an elevated risk of developing contralateral breast cancer, with a cumulative incidence up to 83% for BRCA1 and 62% for BRCA2 carriers^5–7^. Although the efficacy of contralateral RRM for women with *BRCA1/2* mutation has been controversial, many studies have suggested a marked decrease of cancer in the opposite breast^8–10^. Therefore, newly diagnosed breast cancer patients with *BRCA1/2* mutation can consider bilateral mastectomy as a treatment option regarding risk-reducing measures. Decision to undergo RRM needs to consider several factors such as patient’s preference, surgeon’s preference, subtype of breast cancer, clinical stage, family history, and genetic predisposition^11–13^.

In this current study, we investigated whether the timing of identification of *BRCA1/2* mutation might affect surgical decision making. Some studies have shown that genetic diagnosis prior to surgery is associated with RRM^12,14,15^. With growth in the number of genetic testing and the development of sequencing technologies, we further investigated factors that enabled patients to obtain knowledge of *BRCA1/2* mutation status prior to initial surgery. In addition, we analyzed oncologic outcomes of *BRCA1/2* mutation carriers with breast cancer according to types of surgery.

## Methods

We conducted a retrospective review of breast cancer patients tested for BRCA1/2 mutations between January 2008 and November 2019 who underwent curative surgery at Samsung Medical Center. Patients were eligible for this study if they underwent upfront surgery or surgery following neoadjuvant chemotherapy. Genetic testing was conducted at any time in relation to their diagnosis of breast cancer or surgery. They were identified to carry a deleterious BRCA1/2 mutation. We excluded patients who had a history of previous breast cancer, bilateral breast cancer, stage IV disease, those had not undergone a curative breast surgery, and male patients.

Patients with breast cancer were routinely transferred to a dedicated genetic counselor for pre-test counseling and *BRCA1/2* testing on the first visit to our outpatient clinic if the following criteria were met and patients agreed. Criteria for *BRCA1/2* testing were as follows: family history of breast and/or ovarian cancer in first- or second-degree relative, diagnosis of breast cancer at 40 years old or younger, bilateral breast cancer, and multiple primary cancers including breast cancer. Post-test counseling was provided to individuals to inform results of testing and discuss treatment options.

We collected information from registered medical records, including age at breast cancer diagnosis, types of breast surgery, administration of neoadjuvant chemotherapy, pathologic staging, tumor characteristics, types of adjuvant therapy, and patient outcome. Patients were categorized as ‘*BRCA1/2* mutation status known prior to surgery’ if their date of *BRCA1/2* testing result was earlier than the date of surgery and ‘*BRCA1/2* mutation status known after surgery’ if their results were yielded on the day of surgery or after surgery. We additionally calculated turnaround time (TAT) of each testing defined by the time interval between the submission of the patient’s peripheral blood and delivery of the verified result.

*BRCA1/2* testing was performed using genomic DNA isolated from peripheral blood. The sequencing method was initially Sanger sequencing. It was then transitioned to next generation sequencing after September 2016. We classified ‘pathogenic’ or ‘likely pathogenic’ results as positive mutation for *BRCA1/2*, while results of ‘benign’, ‘likely benign’, or ‘uncertain significance’ were classified as negative mutations^16^.

Comparison of patients with *BRCA1/2* mutation identified prior to surgery and those after surgery was performed using the Chi-Square test or Fischer’s exact test for categorical variables and Wilcoxon rank sum test for continuous variables. Multivariable analysis using logistic regression with backward variable selection was conducted to identify factors associated with *BRCA1/2* mutation status known prior to surgery. Kaplan–Meier curves were used to analyze recurrence-free survival (RFS) and overall survival (OS). Log-rank tests were used to compare survival outcomes between patients who underwent ipsilateral surgery only and patients who underwent contralateral RRM. Cumulative incidence function plot was used to analyze the 5-year cumulative incidence of contralateral breast cancer. Gray’s test was used to compare outcomes between the two groups. All statistical analyses were executed using SAS version 9.4 (SAS Institute, Cary, NC, USA) and R 3.6.1 (Vienna, Austria; http://www.R-project.org/). A *P*-value of < 0.05 was considered statistically significant.

All study procedures were conducted in accordance with the Declaration of Helsinki and were approved by the Institutional Review Board (IRB) of Samsung Medical Center, South Korea (IRB File No. 2020-12-145). The requirement for informed consent was waived by the IRB of Samsung Medical Center because of the retrospective nature of this study.

## Results

### Patient characteristics

Among 3950 breast cancer patients who were tested for *BRCA1/2* mutation between January 2008 and November 2019 in Samsung Medical Center, 486 patients were *BRCA1/2* mutation carriers. A total of 344 patients were eligible for the final analysis. Of the final cohort, 140 (40.7%) patients had their *BRCA1/2* mutation status identified prior to surgery (Group 1), while 204 (59.3%) were aware of their mutation status after surgery (Group 2) (Table 1). Women in Group 1 were younger (37.5 years vs 42.0 years, *p* < 0.001) with shorter TAT of *BRCA1/2* mutation testing compared to Group 2 (21 days vs 29 days, *p* < 0.001). Almost two thirds (60.7%) of subjects in Group 1 received neoadjuvant chemotherapy as primary treatment, whereas 89.2% of patients in the Group 2 underwent surgery as primary treatment (*p* < 0.001). Higher clinical T stage and N stage were observed in Group 1 than in Group 2. Additionally, subjects in Group 1 were less likely to be treated with adjuvant chemotherapy (42.9% vs 73.4%) and radiotherapy (60.7% vs 80.8%). Proportions of *BRCA1/2* mutations, histology, and biologic subtype were not significantly different between the two groups. Out of 14 patients with HER2 positive receptors, 12 received anti-HER2 therapy, and the remaining two with ductal carcinoma in situ did not receive such therapy.

**Table 1:** Clinicopathological and treatment characteristics of patients.

Characteristic	BRCA status known prior to surgery (Group 1)	BRCA status known after surgery (Group 2)	Total	*p* value
**Age (years)**				0.027
Median, ranges	37.5 (23.0–67.0)	42.0 (25.0–75.0)	39.0 (23.0–75.0)	
≤ 40	89 (63.6%)	95 (46.6%)	184 (53.5%)	0.002
> 40	51 (36.4%)	109 (53.4%)	160 (46.5%)	
**Tunaround time of BRCA1/2 testing (days)**				< 0.001
Median, ranges	21 (8–85)	29 (8–97)	27.0 (8–97)	
**Family history of breast cancer**				0.605
Yes	89 (64.0%)	125 (61.3%)	214 (62.4%)	
No	50 (36.0%)	79 (38.7%)	129 (37.6%)	
Missing data	1			
**BRCA1/2 results**				0.096
BRCA1 mutation	76 (54.3%)	92 (45.1%)	168 (48.8%)	
BRCA2 mutation	63 (45.0%)	110 (53.9%)	173 (50.3%)	
BRCA1 and BRCA2 mutations	1 (0.7%)	2 (1.0%)	3 (0.9%)	
**Histology**				0.783
Invasive ductal carcinoma	127 (90.7%)	183 (89.7%)	310 (90.1%)	
Invasive lobular carcinoma	3 (2.1%)	3 (1.5%)	6 (1.7%)	
Ductal carcinoma in situ	4 (2.9%)	11 (5.4%)	15 (4.4%)	
Others	6 (4.3%)	7 (3.4%)	13 (3.8%)	
**Ki-67**				0.319
> 20%	111 (79.9%)	152 (75.2%)	263 (77.1%)	
≤ 20%	28 (20.1%)	50 (24.8%)	78 (22.9%)	
Missing data	1	2	3	
**Biologic subtype**				0.847
HR + /HER2−	75 (54.0%)	105 (52.5%)	180 (53.1%)	
HR + /HER2 +	2 (1.4%)	6 (3.0%)	8 (2.4%)	
HR−/HER2 +	4 (2.9%)	2 (1.0%)	6 (1.8%)	
HR−/HER2−	58 (41.7%)	87 (43.5%)	145 (42.8%)	
Missing data	1	4	5	
**Clinical T stage**				< 0.001
T0	4 (2.9%)	12 (5.9%)	16 (4.7%)	
T1	39 (28.1%)	100 (49.0%)	139 (40.5%)	
T2	66 (47.5%)	78 (38.2%)	144 (42.0%)	
T3	28 (20.1%)	14 (6.9%)	42 (12.2%)	
T4	2 (1.4%)	0 (0.0%)	2 (0.6%)	
Missing data	1	0	1	
**Clinical N stage**				< 0.001
N0	67 (48.2%)	136 (66.7%)	203 (59.2%)	
N1	26 (18.7%)	41 (20.1%)	67 (19.5%)	
N2	27 (19.4%)	20 (9.8%)	47 (13.7%)	
N3	19 (13.7%)	7 (3.4%)	26 (7.6%)	
No axillary surgery	1	0	1	
**Primary treatment**				< 0.001
Neoadjuvant chemotherapy followed by surgery	85 (60.7%)	22 (10.8%)	107 (31.1%)	
Upfront surgery	55 (39.3%)	182 (89.2%)	237 (68.9%)	
**Pathologic T stage**^**a**^				0.009
T0	33 (23.6%)	13 (6.4%)	46 (13.4%)	
T1	65 (46.4%)	111 (54.4%)	176 (51.2%)	
T2	31 (22.1%)	74 (36.3%)	105 (30.5%)	
T3	11 (7.9%)	6 (2.9%)	17 (4.9%)	
**Pathologic N stage**^**a**^				
N0	99 (70.7%)	132 (67.3%)	231 (68.8%)	0.760
N1	29 (20.7%)	48 (24.5%)	77 (22.9%)	
N2	9 (6.4%)	13 (6.6%)	22 (6.5%)	
N3	3 (2.1%)	3 (1.5%)	6 (1.8%)	
No axillary surgery	0	8	8	
**Adjuvant chemotherapy**				< 0.001
Yes	60 (42.9%)	149 (73.4%)	209 (60.9%)	
No	80 (57.1%)	54 (26.6%)	134 (39.1%)	
Missing data	0	1	1	
**Adjuvant radiotherapy**				< 0.001
Yes	85 (60.7%)	164 (80.8%)	249 (72.6%)	
No	55 (39.3%)	39 (19.2%)	94 (27.4%)	
Missing data	0	1	1	
**Adjuvant hormone therapy**				0.975
Yes	78 (55.7%)	114 (55.9%)	192 (55.8%)	
No	62 (44.3%)	90 (44.1%)	152 (44.2%)	
Total	140 (40.7%)	204 (59.3%)	344 (100.0%)	

*HR* hormone receptor, *HER2* human epidermal growth factor receptor 2.

^a^Pathologic T stage and pathologic N stage include post neoadjuvant therapy pathological category.

### Types of surgery according to preoperative knowledge of BRCA1/2 mutations

**Figure 1:**
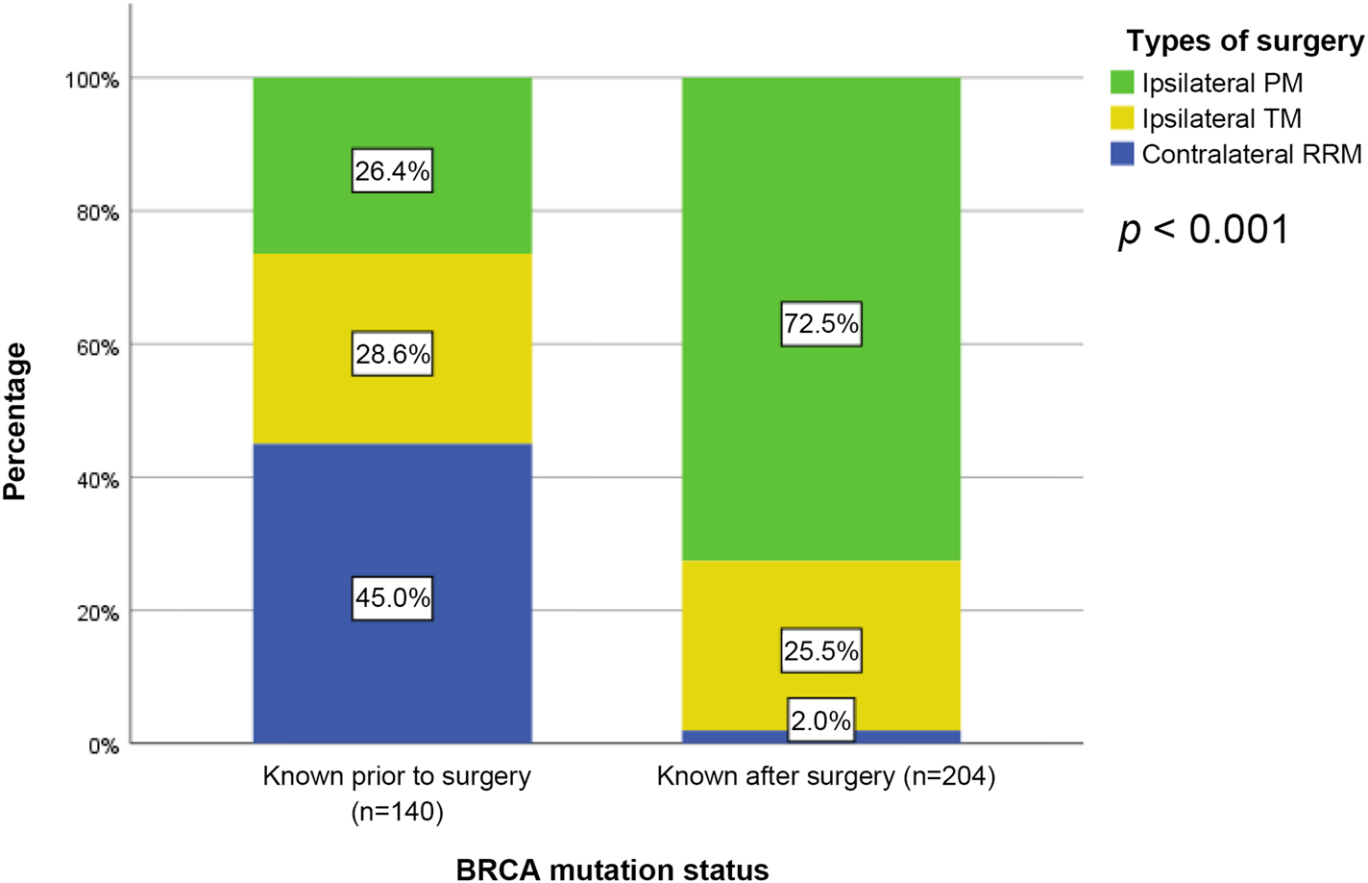
Types of surgery according to the timing of identification of BRCA1/2 mutation status. *PM* partial mastectomy, *TM* total mastectomy, *RRM* risk reducing mastectomy.

As shown in Fig. 1, contralateral RRM was performed for 45.0% (63/140) of patients with *BRCA1/2* mutation identified prior to surgery. The rate was significantly (*p* < 0.001) lower for patients with *BRCA1/2* mutation identified after surgery, of which only 2.0% (4/204) chose contralateral RRM as initial surgery whereas 72.5% (148/204) elected ipsilateral partial mastectomy and 25.5% (52/204) elected ipsilateral total mastectomy. Out of the whole cohort, 13 patients eventually underwent bilateral mastectomy after their initial surgery, including 8 patients whose *BRCA1/2* mutation were identified after the initial surgery, 1 patient who was a known *BRCA1/2* mutation carrier prior to initial surgery, and 4 who developed contralateral breast cancer.

### Factors associated with BRCA1/2 mutation status known prior to surgery

Multivariable analysis was conducted using factors that were significant in univariable analysis, including patient age, TAT of *BRCA1/2* testing, higher clinical T, N stage, and neoadjuvant chemotherapy (Table 2). Patients were significantly more likely to be informed of BRCA mutation status if they received neoadjuvant chemotherapy (OR: 22.43; 95% CI: 11.26–44.64) or their TAT of testing was significantly associated with *BRCA1/2* mutation status known prior to surgery.

**Table 2:** Multivariable analysis of factors associated with *BRCA1/2* mutation status known prior to surgery.

Variable	Odds ratio	95% CI	*p* value
Age	1.00	0.97–1.03	0.855
Tunaround time of BRCA1/2 testing	0.94	0.92–0.96	< 0.001
**Primary treatment**			
Upfront surgery (ref.)			
Neoadjuvant chemotherapy	22.43	11.26–44.64	< 0.001
**Clinical T stage**			
cT0 (ref.)			
cT1	1.29	0.35–4.75	0.703
cT2	1.48	0.38–5.72	0.568
cT3	2.00	0.32–12.12	0.460
cT4	NA	NA	
**Clinical N stage**			
cN0 (ref.)			
cN1	1.31	0.62–2.75	0.483
cN2	2.06	0.72–5.88	0.177
cN3	0.69	0.14–3.46	0.654

Multivariable analysis was conducted by logistic regression with backward variable selection.

### Oncologic outcomes of BRCA1/2 mutation carriers with breast cancer according to types of initial surgery

**Figure 2:**
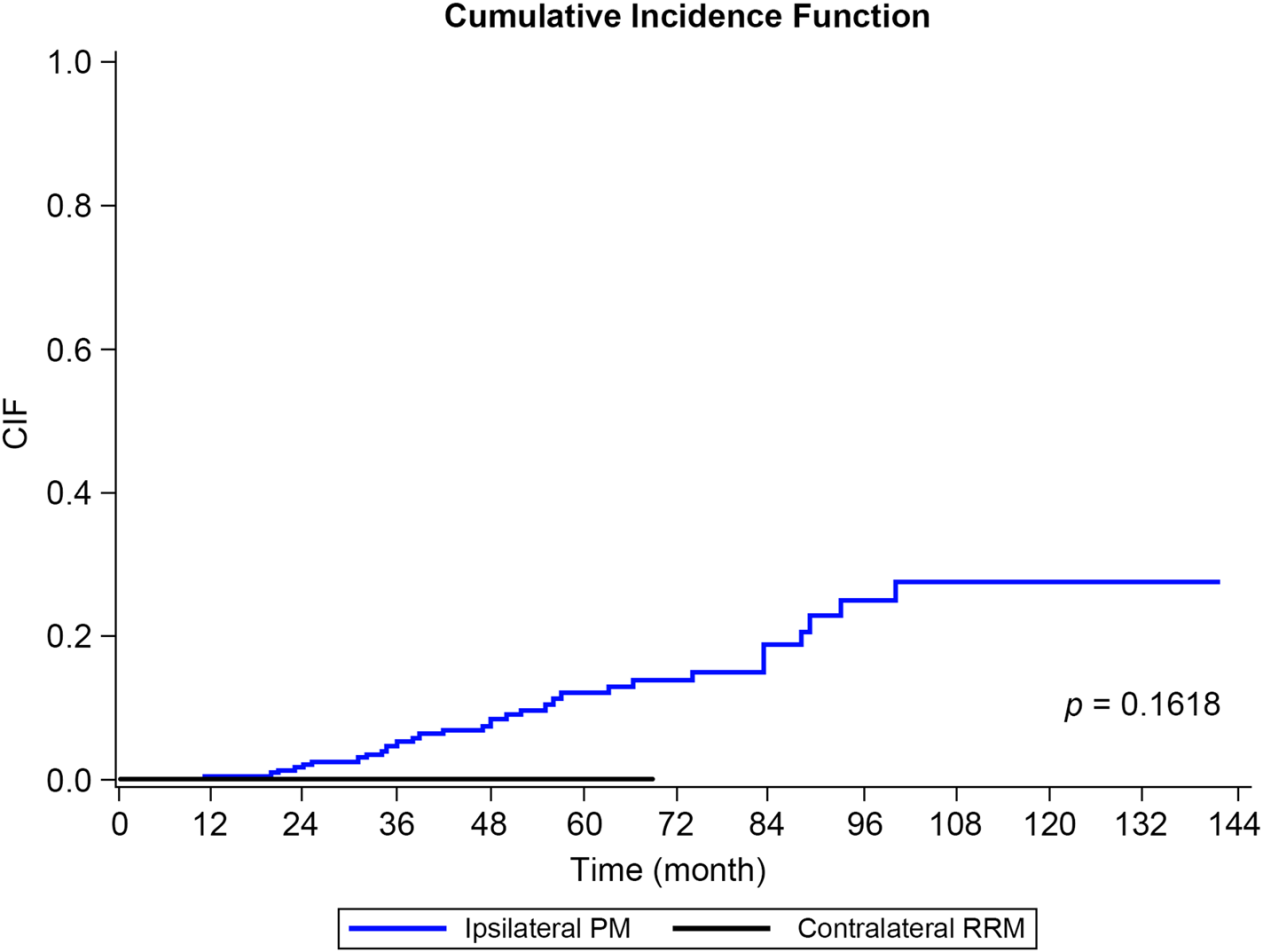
Cumulative incidence function plot of contralateral breast cancer according to types of surgery. *PM* partial mastectomy, *RRM* risk reducing mastectomy.

Of 344 patients in the cohort, 67 (19.5%) patients underwent contralateral RRM and 277 (80.5%) patients underwent ipsilateral surgery as initial surgery. The median follow-up was 41 months for the total patients, 46 months for the ipsilateral surgery only group, and 15 months for the contralateral RRM group. There was no significant difference in overall survival (log-rank *p* = 0.938) or disease-free survival (log-rank *p* = 0.864) between the two surgery groups. No patient in the contralateral RRM group experienced contralateral breast cancer during the follow-up. However, 10.8% (30/277) of the ipsilateral surgery-only group developed contralateral breast cancer. The estimated 5-year cumulative incidence of contralateral breast cancer was 12.1% (95% CI 7.7–17.7%) for the ipsilateral surgery-only group. This incidence was not available for the contralateral RRM group because contralateral breast cancer did not occur (Fig. 2).

### Median TATs for *BRCA1/2* testing

**Figure 3:**
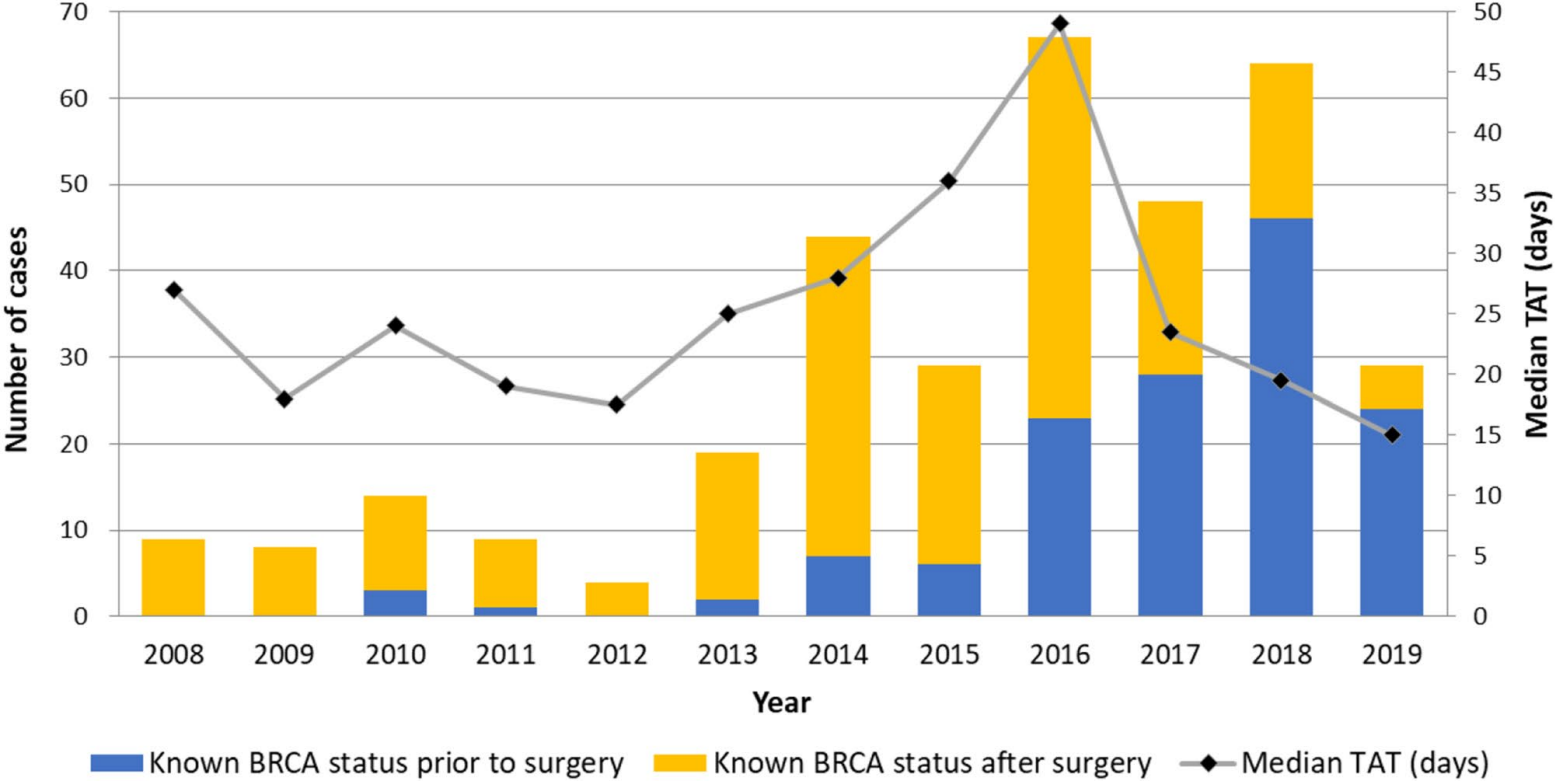
Bar and line graph showing number of cases tested positive for BRCA1/2 who underwent curative breast cancer surgery and median turnaround time for BRCA1/2 testing in Samsung Medical Center. *TAT* turnaround time.

Annual number of cases tested positive for *BRCA1/2* mutation who underwent curative breast cancer surgery and median TATs for *BRCA1/2* testing in Samsung Medical Center is shown in Fig. 3. A definite increase in the *BRCA1/2* mutation carriers was observed in 2014. Since then, proportions of patients with known *BRCA1/2* status prior to surgery to *BRCA1/2* mutation carriers have steadily increased. The bold line indicates the median value of TATs. The median TAT dropped from 49 days in 2016 to 15 days in 2019.

## Discussion

In the present study, concomitant contralateral RRM was significantly more prevalent in breast cancer patients whose *BRCA1/2* mutations were identified prior to surgery. We found that most patients underwent either ipsilateral partial mastectomy or ipsilateral total mastectomy when their mutation status was unknown at initial surgery. This is line with earlier studies reporting that preoperative genetic testing impacts surgical decision making in favor of RRM^12,14,15,17,18^. We additionally demonstrated several factors that enabled patients to gain knowledge about their *BRCA1/2* mutation status prior to initial surgery. Although not statistically significant, we observed a higher risk of developing contralateral breast cancer for *BRCA1/2* mutation carriers who underwent ipsilateral surgery-only compared to those who underwent contralateral RRM. Preoperative testing and diagnosis of *BRCA1/2* mutation provided opportunities for patients to engage in risk reducing measures at the time of primary surgery.

Short TATs of sequencing for *BRCA1/2* mutation allowed patients to receive their results before the initial surgery. A study has assessed TATs of *BRCA1/2* testing among ovarian cancer patients and revealed that amount of time it takes from initial genetic counseling to receipt of testing results is mainly driven by sample processing time, with median TATs varied among countries (2.7 weeks, 14.6 weeks, and 5.9 weeks in the US, Italy, and Spain, respectively)^19^. In our study, the median TAT in the group that mutation status was known prior to surgery was 21 days, whereas the median TAT in the group that mutation status was identified after surgery was 30 days. Traditionally, genetic testing to identify *BRCA1/2* mutation has been conducted by Sanger sequencing. Sanger sequencing analysis generally requires 4 to 6 weeks of TAT in South Korea, making it difficult for patients to obtain genetic information preoperatively^20^. The development of next-generation sequencing (NGS) and its application for detecting genetic mutations have significantly shortened the analysis time and lowered the expenditure. Our institution adopted NGS for identifying *BRCA1/2* mutations in late 2016. We have observed reduced TATs since then, proven by decreasing line graph shown in Fig. 3. Our previous retrospective study included 164 *BRCA1/2* mutation carriers who underwent curative breast cancer surgery between 2004 and 2015 and identified only 15 patients with known BRCA mutation prior to surgery^15^. The current study expanded the inclusion period until late 2019. The number of patients with known *BRCA1/2* mutation status prior to surgery was increased up to 140. Recent increase of patients with *BRCA1/2* mutation status identified prior to surgery could be explained by the application of NGS on *BRCA1/2* mutation testing which reduced TATs and increased the use of neoadjuvant chemotherapy.

Our study demonstrates that undergoing neoadjuvant chemotherapy has great odds of gaining knowledge of *BRCA1/2* mutation status prior to surgery. It allows the time it takes for genetic testing to yield results while patients are treated. However, out of 107 patients who underwent neoadjuvant chemotherapy, 22 patients did not receive results preoperatively. This was mainly due to late identification of patients with high risk of hereditary breast cancer or delayed prescription of *BRCA1/2* mutation testing.

Trained personnel, training programs, and formal accreditation processes are essential for accessibility to genetic counselling and testing. South Korea has implemented a national program for genetic counselling and genetic testing through national healthcare system, which has eventually increased proportions of genetic testing uptake to breast cancer incidence of 7.7%, the highest in Asia^21^. A prior study has indicated that early detection and genetic referral of patients who meet guidelines for *BRCA1/2* testing are important for receiving testing results prior to surgery^12^. According to the American BRCA Outcomes and Utilization of Testing (ABOUT) Study^22^, lack of physician recommendation is the most commonly reported reason for not receiving genetic counseling. In our clinic, genetic counseling and testing are offered to patients with high risk of hereditary breast cancer on the first day of visit following breast cancer diagnosis. A concept of rapid genetic counseling and testing (RGCT) in newly diagnosed breast cancer patients is being emphasized. In order to maximize the likelihood that test results can be used for surgical decision, surgeons need to provide genetic testing at the point of care. They could even delay primary surgery slightly for a small subset of high-risk patients^23–25^.

In this study, contralateral RRM demonstrated no survival benefit for *BRCA1/2* mutation carriers with breast cancer. This is in line with a meta-analysis by Valanchis^4^, stating that there is no significant difference in breast-cancer-specific survival between *BRCA1/2* carriers who undergo contralateral RRM and breast conserving therapy. However, mortality benefit from preventing contralateral second primary cancer is likely to be apparent after an extended period^26,27^. Metcalf et al.^26^ have noted a 48% reduction of death from breast cancer in *BRCA1/2* mutation carriers who undergo contralateral RRM compared to those who undergo ipsilateral mastectomy after a 20-year follow-up.

The greater risk of developing contralateral breast cancer in *BRCA1/2* mutation carriers compared to women with sporadic cancer supports the necessity of RRM for the unaffected side of breast for *BRCA1/2* mutation carriers. The cumulative incidence for mutation carriers vs. sporadic cases is 20% vs. 2% at 5 years and 42% vs 9% at 12 years^28–30^. A previous case–control study conducted in our center has shown 4.0% of metachronous contralateral breast cancer recurrence in *BRCA1/2*-negative patients with risk factors for hereditary breast and/or ovarian cancer^31^. However, the current study showed 8.4% of metachronous contralateral breast cancer recurrence in patients with *BRCA1/2* mutations.

Several studies have concluded that contralateral RRM could reduce the risk among *BRCA1/2* mutation carriers with a history of breast cancer^8,14,32,33^. Van Sprundel et al.^8^ have reported a reduction of contralateral breast cancer risk by 91%, independent of the effect of bilateral prophylactic oophorectomy. Patients in our cohort who underwent contralateral RRM did not develop any contralateral breast cancer. The 5-year cumulative incidence of contralateral breast cancer patients who underwent ipsilateral surgery only was 12.1%. However, the difference in the risk of contralateral breast cancer between those two groups was insignificant. This might be due to a follow-up time as short as 15 months for the contralateral RRM group. The Korean National Health Insurance coverage for contralateral breast surgery in breast cancer patients with *BRCA1/2* mutation carrier was initiated in March 2017 and the first patient in our cohort to receive contralateral RRM was in April 2014. These conditions might have resulted in a relatively short median follow-up time for the contralateral RRM group.

Decision to undergo RRM is a complex process, which involves weighing the benefits and harms. Of 140 patients with *BRCA1/2* mutation status identified prior to surgery, 77 (55%) did not choose RRM as their initial surgery. Previous studies stated that reasons for not opting RRM include its lack of survival benefit, increased operative morbidity, potential to delay adjuvant therapy if complications occur, and possible poor cosmetic outcome^34,35^. Benefits for undergoing RRM include reducing the risk of contralateral breast cancer and possibilities of avoiding additional treatment. Patients who were unaware of their *BRCA1/2* status and underwent partial mastectomy received radiotherapy. If they eventually undergo mastectomy and reconstruction afterwards, previous radiation could elicit complications. Of 30 patients who developed contralateral breast cancer in our cohort, 11 (36.7%) were triple-negative breast cancer. All 30 patients underwent subsequent curative surgery, 13 (43.3%) received chemotherapy, and 15 (50.0%) received radiotherapy. These additional treatments could have been spared if *BRCA1/2* carriers with unilateral breast cancer underwent RRM at the time of initial surgery. Furthermore, although intensive surveillance could be opted for *BRCA2* carriers due to low mortality, women electing for RRM want to avoid the lifelong distress related to intensive surveillance^36^.

This study has several limitations. First, this was a retrospective analysis of patients treated at a single institution, which could limit the generalizability of our findings. Second, the criterion used to categorize patients was ‘date of *BRCA1/2* testing result’, which could lead to overestimation of patients with *BRCA1/2* mutation status known prior to surgery. Thirteen patients had no actual record about delivery of mutation status prior to surgery. However, all patients in the cohort received pre-test counseling. In addition, we routinely informed *BRCA1/2* testing result even on the day before surgery. Third, factors that could affect surgical decision to undergo RRM were not analyzed. We demonstrated that reduced TATs and treating with neoadjuvant chemotherapy enabled patients to be aware of *BRCA1/2* mutation status preoperatively. These factors could potentially affect surgical decision-making. However, their direct associations with increased RRM were not proved. Lastly, our oncologic outcomes have limitation for interpretation due to the short follow-up time for the ipsilateral surgery-only group and lack of adjustment for factors that affect outcomes such as age and use of adjuvant therapy. Further investigation on oncologic outcomes with an extended period of follow-up and adjustment for clinical variables is required. These limitations were countered by the significance of present study. We demonstrated that long TAT of gene testing as well as lack of systemized genetic counseling, delay in referral of patients to genetic counselor impeded patients’ preoperative awareness of *BRCA1/2* mutation status. As far as we know, it is the first to present such results in Asia.

## Conclusions

In conclusion, our findings demonstrate that preoperative identification of *BRCA1/2* mutation could affect surgical decision-making for breast cancer patients in favor of contralateral RRM. With the application of NGS for *BRCA1/2* testing and the use of neoadjuvant chemotherapy, more patients are able to acquire their mutation status prior to initial surgery. Although undergoing contralateral RRM may not guarantee improved survival, it could reduce contralateral breast cancer risk, contribute to the reduction of additional future surgeries, unnecessary adjuvant radiation, and improve patient satisfaction. It is important that clinicians provide genetic counseling and testing at the time of diagnosis in order to inform patients of their *BRCA1/2* mutation status prior to surgery and provide them with optimal treatment options.

## Data Availability

The datasets generated during and/or analyzed during the current study are available from the corresponding author on reasonable request.
